# Time course-changes in phosphatidylcholine profile during oxidative modification of low-density lipoprotein

**DOI:** 10.1186/1476-511X-13-48

**Published:** 2014-03-14

**Authors:** Naoko Sasabe, Yuka Keyamura, Takashi Obama, Nozomi Inoue, Yukihiro Masuko, Yu Igarashi, Toshihiro Aiuchi, Rina Kato, Tomohiro Yamaguchi, Hiroshi Kuwata, Sanju Iwamoto, Akira Miyazaki, Shuntaro Hara, Tomohiro Yoshikawa, Hiroyuki Itabe

**Affiliations:** 1 Division of Biological Chemistry, Department of Molecular Biology, Showa University School of Pharmacy, Tokyo, Japan; 2 Free Radical Research Project, Otsuka Pharmaceutical Co. Ltd., Tokushima, Japan; 3 Division of Health Chemistry, Department of Healthcare and Regulatory Sciences, Showa University School of Pharmacy, Tokyo, Japan; 4 Department of Biochemistry, Showa University School of Medicine, Tokyo, Japan

**Keywords:** Oxidized LDL, PC molecular species, LC-MS/MS, Oxidized PC, PAF-AH, Pefabloc, lysoPC, PONPC, Rabbit LDL

## Abstract

**Background:**

Oxidized phosphatidylcholines (oxPC) and lysophosphatidylcholine (lysoPC) generated during the formation of oxidized low-density lipoprotein (oxLDL) are involved in atherosclerotic lesion development. We investigated the time course-changes in phosphatidylcholine (PC) molecular species during oxidation of LDL to determine how those atherogenic PCs are produced.

**Methods:**

Human and rabbit LDLs were pretreated with or without a selective platelet-activating factor acetylhydrolase (PAF-AH) inhibitor. LDL was oxidized by incubation with copper sulfate, and PC profiles were analyzed by liquid chromatography-tandem mass spectrometry.

**Results:**

When human LDL was oxidized, the peak areas for polyunsaturated fatty acid (PUFA)-containing PC species dramatically decreased after a short lag period, concomitantly lysoPC species increased sharply. Although a variety of oxPC species containing oxidized fatty acyl groups or cleaved acyl chains are formed during LDL oxidation, only a few oxPC products accumulated in oxLDL: 1-palmitoyl-2-(9-oxo-nonanoyl) PC and long-chain oxPC with two double bonds. Pretreatment of LDL with the PAF-AH inhibitor greatly reduced lysoPC production while it had no effect on lipid peroxidation reactions and oxPC profiles. Rabbit LDL, which has a different composition of PC molecular species and needs a longer time to reach achieve full oxidation than human LDL, also accumulated lysoPC during oxidation. The increase in lysoPC in rabbit oxLDL was suppressed by pretreatment with the PAF-AH inhibitor. The major oxPC species formed in rabbit oxLDL were almost the same as human oxLDL.

**Conclusions:**

These results suggest that lysoPC species are the major products and PAF-AH activity is crucial for lysoPC generation during oxidation of LDL. The oxPC species accumulated are limited when LDL is oxidized with copper ion *in vitro*.

## Background

Oxidative modification of low-density lipoprotein (LDL) is involved in atherosclerotic lesion development [1-3]. Studies have shown significant increases in plasma circulating oxidized LDL (oxLDL) in patients with cardiovascular diseases [4-8]. LDL consists of phospholipids, cholesterol ester, triacylglycerol, and an apolipoprotein B (apoB) together with several minor constituents, so that oxLDL may contain a large variety of oxidized products and modified molecules [9-11].

Many biological and pathological activities of oxLDL have been investigated, and oxidized phosphatidylcholine (oxPC) and lysophosphatidylcholine (lysoPC) formed during oxidation of LDL are thought to be responsible for some oxLDL functions. OxPC is a mixture of diverse products characterized by various functional groups, including both long chain acyl groups with hydroperoxide or hydroxyl function and truncated short chain acyl groups. Macrophage scavenger receptors take up oxLDL leading to foam cell formation, where oxPC is involved in recognition by scavenger receptors [12,13]. OxPC could act as a peroxisome proliferator activating receptor (PPAR)-γ activator to induce inflammatory and metabolic responses [14]. It promotes chemokine production in endothelial cells [15,16] and proliferation and calcification of smooth muscle cells [17]. In addition, oxPC induces inflammatory reactions by acting as an environmental pathogen mimetic [18]. OxPC, especially PC hydroperoxides (PCOOH), increases macrophage adhesion to ICAM-1 through reorganization of microfilaments [19]. LysoPC is another atherogenic metabolite formed in oxLDL that shows many biological effects including attenuation of endothelial functions and proliferation of smooth muscle cells [20,21].

OxPC is further modified chemically or enzymatically in plasma. Several enzymes metabolize oxPC, including phospholipid hydroperoxide glutathione peroxidase (PHGPx) [22], secretory phospholipase A_2_ type IIA (sPLA_2_-IIA) [23], lecithin-cholesterol acyltransferase (LCAT) [24], and platelet-activating factor acetyl hydrolase (PAF-AH/Lp-PLA_2_) [25]. These enzyme activities make oxidized PC profiles more complex.

PAF-AH is a unique plasma phospholipase that hydrolyzes PC species containing short and/or hydrophilic acyl chains as substrates in addition to PAF itself [25,26]. It is considered that PAF-AH contributes substantially to the detoxification of pro-atherogenic oxPC products into lysoPC. Steinbrecher reported that the phospholipase A_2_ activity for short-chain PC substrates in human LDL was not separated from PAF-AH [27]. The enzyme associates with lipoprotein particles, LDL and high-density lipoprotein (HDL), in human plasma [25] and is found primarily in association with HDL in rabbit and rodent plasma [28,29]. Because PAF-AH has an active serine residue in its catalytic center, enzyme activity is inhibited by serine-protease inhibitors such as phenylmethylsulfonyl fluoride (PMSF). Pefabloc, a sufonylfluoride compound similar to PMSF, does not inhibit paraoxonase-1, LCAT, or venom phospholipase A_2,_ has been used as a selective inhibitor of PAF-AH [30-32].

Although the pathological impact of oxLDL is recognized, neither the complete structure of oxLDL nor the molecular process of oxLDL generation has been fully determined. Recently liquid chromatography-tandem mass spectrometry (LC-MS/MS) has been applied to the comprehensive analysis PC molecular species including oxidized products [33-35]. Rabbits have been used for diet-induced atherosclerosis experiments, although many animals including rodents are not susceptible to atherosclerosis without genetic manipulation. Checking PC profiles of human and rabbit oxLDLs could give an idea for atherogenic oxPC products. In the present study, we investigated the PC profile changes during copper-induced oxidation of LDL to elucidate the process of PC modification by LC-MS/MS. We also investigated the contribution of PAF-AH to PC profile changes using a PAF-AH inhibitor and rabbit LDL in addition to human LDL.

## Results

### PC profile change in human LDL during oxidation

**Figure 1:**
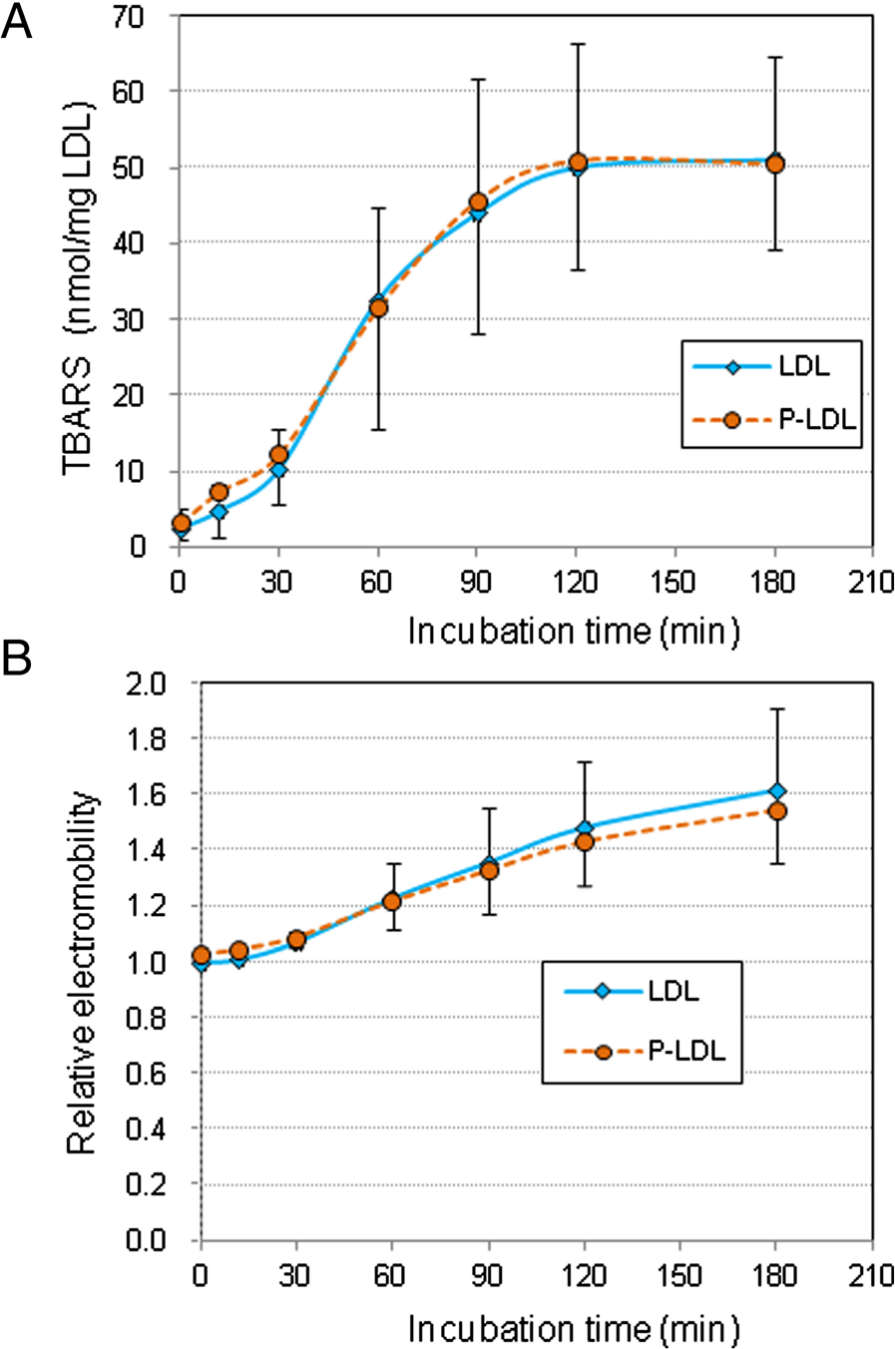
**Time course of copper-induced oxidation of human LDL.** Human LDL (0.2 mg/mL) treated either with or without a PAF-AH inhibitor, pefabloc, was incubated with copper sulfate (5 μM) at 37°C for up to 3 h. The oxidative change of LDL during the incubation was evaluated by production of TBARS **(A)** and relative mobility on agarose gel electrophoresis **(B)**. Values indicated are mean ± SD of 8–10 experiments.

Human LDL treated with or without pefabloc, a PAF-AH inhibitor, was incubated with copper sulfate for up to 3 h and the profile of PC molecular species was determined. When human LDL was pretreated with pefabloc for 30 min, 98% of the PAF-AH activity was inhibited, as determined using 2-thio-PAF as substrate. Pretreatment of LDL with pefabloc showed no differences in increases of thiobarbituric acid-reactive substances (TBARS) and electromobility shift in agarose gel electrophoresis during oxidation (Figure 1), suggesting that peroxidation reactions in the LDL proceed equally in pefabloc-treated LDL and non-treated LDL.

**Figure 2:**
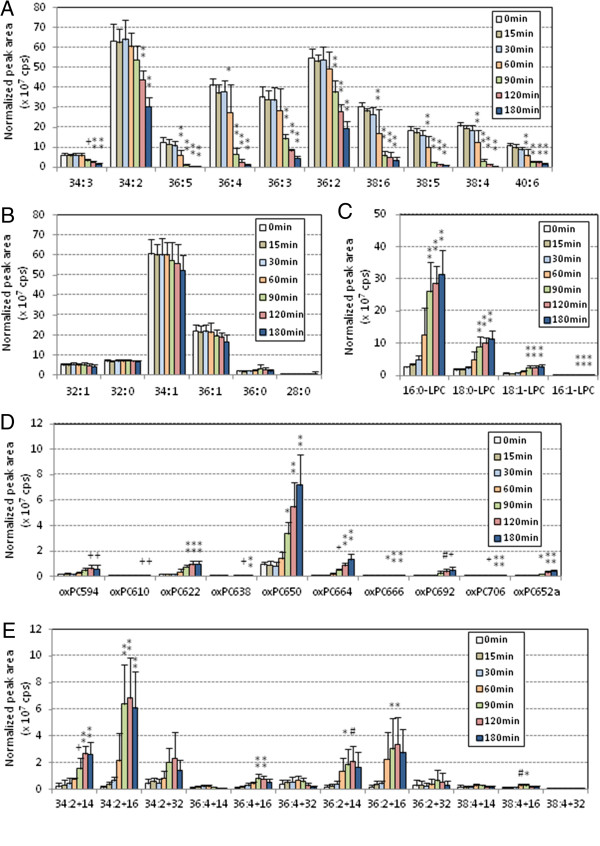
**PC profile changes during copper-induced oxidation of human LDL.** Human LDL (0.2 mg/mL) was incubated with copper sulfate (5 μM) at 37°C for up to 3 h. Lipids were extracted by the Bligh and Dyer method from 10 μg of oxLDL after addition of 20 pmol of didecanoyl PC as internal standard. Total lipid extract was subjected to LC-MS/MS analysis to detect 42 PC and modified PC molecular species by MRM mode, and they were classified into five categories: PUFA-PC **(A)**, S/MUFA-PC **(B)**, lysoPC **(C)**, cleaved-chain oxPC **(D)** and long-chain oxPC **(E)**. The peak area (cps) for each species was normalized based on the peak areas of internal standard, and the means and SD were calculated from six independent preparations. Statistical significance of comparison with the sample without incubation (0 min) was calculated by ANOVA; *, *p* < 0.05; #, *p* < 0.01; +, *p* < 0.005; **, *p* < 0.001.

Using LC-MS/MS with multiple-reaction monitoring (MRM), 42 PC species with selected m/z values were detected simultaneously in each oxLDL sample (Table 1). These PC species were classified into five groups: PC containing polyunsaturated fatty acids (PUFA-PC), PC with only saturated or monounsaturated fatty acids (S/MUFA-PC), lysoPC, oxPC with truncated acyl chains (cleaved oxPC), and oxPC with oxidized acyl chains (long chain oxPC). Figure 2 shows the changes in peak area of each PC species during copper-induced oxidation of human LDL. PUFA-PC species did not change for the first 30 min, then decreased dramatically (Figure 2A), whereas S/MUFA-PC did not change throughout the incubation (Figure 2B). LysoPC species, primarily 16:0-LPC and 18:0-LPC, increased gradually until 30 min and then jumped up during copper-induce oxidation (Figure 2C). The amounts of oxPC increased slightly throughout the incubation period. Many oxPC species were formed, but the major product appeared to be 1-palmitoyl-2-(9-oxo-nonanoyl) PC (PONPC; m/z = 650.6) (Figure 2D). Among the long-chain oxPC, species with two double bonds, derived from linoleate-containing PCs, accumulated during the incubation period. In contrast, those with four double bonds, likely derived from arachidonate-containing PCs, did not accumulate or even decreased after 1 h incubation (Figure 2E).

**Table 1:** List of 42 PC species monitored by LC-MS/MS

**m/z**	**Molecular species**	**m/z**	**Molecular species**
S/MUFA-PC		Long chain oxPC	
732.6	32:1	772.6	34:2 + 14
734.6	32:0	774.6	34:2 + 16
760.6	34:1*	790.6	34:2 + 32
788.6	36:1	796.6	36:4 + 14
790.6	36:0	798.6	36:4 + 16
678.4	28:0*	814.6	36:4 + 32
		800.6	36:2 + 14
PUFA-PC		802.6	36:2 + 16
756.6	34:3	818.6	36:2 + 32
758.6	34:2*	824.6	38:4 + 14
780.6	36:5	826.6	38:4 + 16
782.6	36:4*	842.6	38:4 + 32
784.6	36:3		
786.6	36:2	Cleaved oxPC	
806.6	38:6	594.6	1-palmitoyl-2-(5-oxovaleroyl) PC*
808.6	38:5	610.6	1-palmitoyl-2-glutaroyl PC*
810.6	38:4	622.6	1-stearoyl-2-(5-oxovaleroyl) PC
834.6	40:6	638.6	1-stearoyl-2-glutaroyl PC
		650.6	1-palmitoyl-2-(9oxo-nonanoyl)PC*
LysoPC		664.6	1-palmitoyl-2-(5-oxo-octenoyl) PC
496.2	16:0-lysoPC*	666.6	1-palmitoyl-2-azelaoyl PC*
524.2	18:0-lysoPC	692.6	1-stearoyl-2-(5-oxo-octenoyl) PC
494.2	16:1-lysoPC	706.6	1-stearoyl-2-(6-oxo-nonenoyl) PC
522.2	18:1-lysoPC	652.6a	1-O-hexadecyl-2-azelaoyl PC*

In MRM mode, signals corresponding to selected m/z values with a fragment ion of 184 were selectively detected. *PC species identified by comparison with commercially available synthetic standard PC species. The other species are inferred from MS data available in the literature.

**Figure 3:**
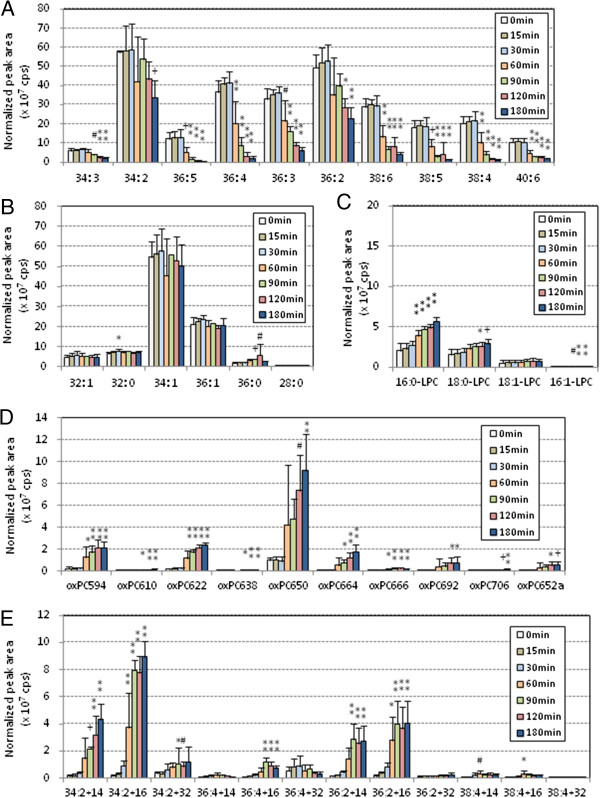
**PC profile changes during copper-induced oxidation of human LDL treated with a PAF-AH inhibitor.** Human LDL was pretreated with pefabloc, a PAF-AH inhibitor, and the treated LDL (0.2 mg/mL) was incubated with copper sulfate (5 μM) at 37°C for up to 3 h. Lipid extraction and LC-MS/MS analysis were performed as in Figure 2. The peak area (cps) for each species was normalized based on the peak areas of internal standard, and means and SD were calculated from six independent preparations. The 42 PC species were classified into five groups: PUFA-PC **(A)**, S/MUFA-PC **(B)**, lysoPC **(C)**, cleaved-chain oxPC **(D)** and long-chain oxPC **(E)**. Statistical significance of comparison with the sample without incubation (0 min) was calculated by ANOVA; *, *p* < 0.05; #, *p* < 0.01; +, *p* < 0.005; **, *p* < 0.001.

Figure 3 shows the PC profile changes during the oxidation of pefabloc treated-human LDL. After the lag time, PUFA-PC species began to decrease at 60 min and S/MUFA-PC did not change throughout the incubation (Figure 3A, B). LysoPC species increased during the incubation, although the peak areas were much smaller than those without pefabloc treatment (Figure 3C). The major oxPC species accumulated were PONPC and long-chain oxPC species with two double bonds (Figure 3D, E) that were also the major species in the absence of pefabloc treatment.

**Figure 4:**
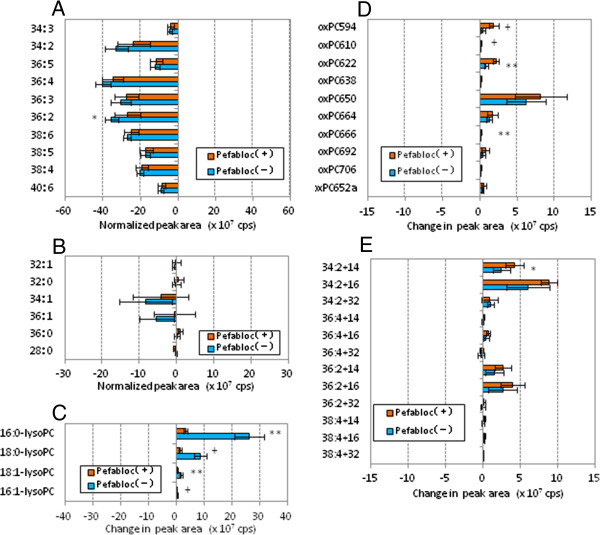
**Effects of PAF-AH inhibitor pretreatment on changes in PC species during oxidation of human LDL.** Human LDL and pefabloc-treated human LDL (0.2 mg/mL) were incubated with copper sulfate (5 μM) at 37°C for 3 h. Lipid extraction and LC-MS/MS analysis were performed, and the change in peak area (cps) during the 3 h oxidation was calculated for each PC species. The 42 PC species were classified into five groups: PUFA-PC **(A)**, S/MUFA-PC **(B)**, lysoPC **(C)**, cleaved-chain oxPC **(D)** and long-chain oxPC **(E)**. Values indicated are means and SD (n = 6). Statistical significance between the samples with and without treatment with PAF-AH inhibitor was calculated by Welch t-test; *, *p* < 0.05; #, *p* < 0.01; +, *p* < 0.005; **, *p* < 0.001.

We calculated the increase or decrease of peak area obtained after the 3 h incubation period for each species to see the effect of pefabloc on the PC profiles (Figure 4). All of the PUFA-PC reduced their peak areas, showing that inhibition of PAF-AH had little effect on changes in PUFA-PC following treatment with copper sulfate (Figure 4A). LysoPC increased their peak areas during the oxidation reaction, and the increase of lysoPC was greatly suppressed when PAF-AH was inhibited (Figure 4C). Pefabloc treatment had limited effect on the increase of cleaved oxPC and long-chain oxPC species, while cleaved oxPC species with five carbon-acyl chains (m/z = 594.2, 610.6 and 622.6) increased slightly (Figure 4D, E).

### PC profiles in rabbit LDL during oxidation

Next, we examined changes in the PC profile in rabbit LDL during oxidation. LDL was prepared from New Zealand white rabbits fed normal diet supplemented with 0.5% cholesterol to increase LDL content in plasma. The PC profile of rabbit LDL is substantially different from that of human LDL. Rabbit LDL is relatively rich S/MUFA-PC and lysoPC. Rabbit LDL was needed a longer incubation than human LDL to reach the maximum oxidation, which may be caused by lower content of PC species with more than five double bonds. PAF-AH activity in the rabbit LDL fraction was estimated to be 2.9 nmol/min/mg, which is 25% of that in human LDL. The PAF-AH activity in the LDL fraction was 6.0% of HDL fraction recovered from the same rabbits, which corresponds to a previous report that PAF-AH is present mostly in HDL in rabbit plasma and very little in LDL [28]. Pretreatment of the rabbit LDL fraction with pefabloc reduced PAF-AH activity by 91%.

**Figure 5:**
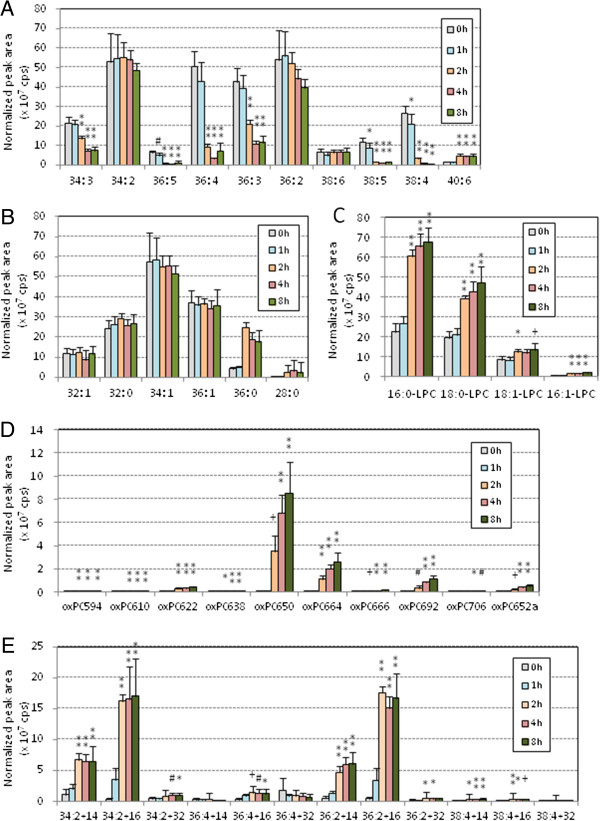
**PC profile changes during copper-induced oxidation of rabbit LDL.** Rabbit LDL (0.1 mg/mL) was incubated with copper sulfate (5 μM) at 37°C for up to 8 h. Lipid extraction and LC-MS/MS analysis were performed as in Figure 2. The peak area (cps) for each species was normalized based on the peak areas of internal standard, and the means and SD were calculated from six independent preparations. The 42 PC and modified PC molecular species by MRM mode, and they were classified into five categories: PUFA-PC **(A)**, S/MUFA-PC **(B)**, lysoPC **(C)**, cleaved-chain oxPC **(D)** and long-chain oxPC **(E)**. Statistical significance of comparison with the sample without incubation (0 h) was calculated by ANOVA; *, *p* < 0.05; #, *p* < 0.01; +, *p* < 0.005; **, *p* < 0.001.

**Figure 6:**
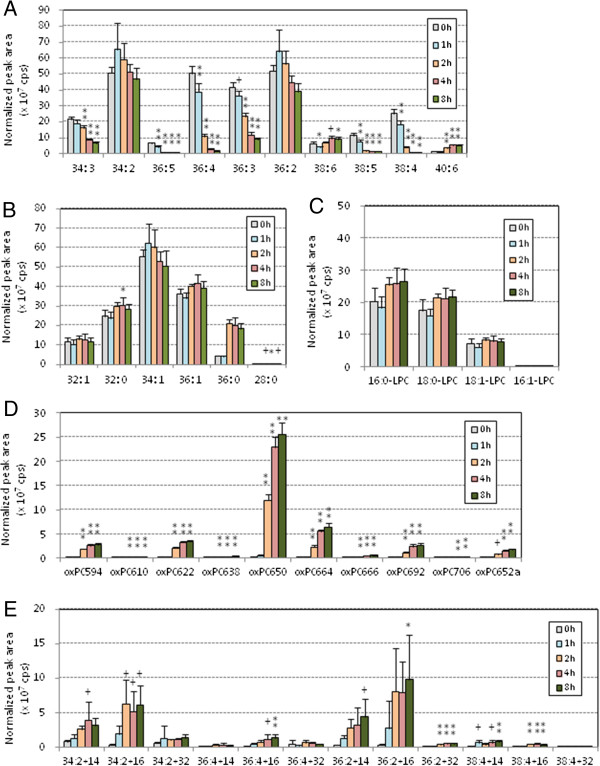
**PC profile changes during copper-induced oxidation of rabbit LDL treated with PAF-AH inhibitor.** Rabbit LDL (0.1 mg/mL) was treated with a PAF-AH inhibitor, pefabloc, and then, incubated with copper sulfate (5 μM) at 37°C for up to 8 h. Lipid extraction and LC-MS/MS analysis were performed as in Figure 2. The peak area (cps) for each species was normalized based on the peak areas of internal standard, and the means and SD were calculated from six independent preparations. The 42 PC species were classified into five groups: PUFA-PC **(A)**, S/MUFA-PC **(B)**, lysoPC **(C)**, cleaved-chain oxPC **(D)** and long-chain oxPC **(E)**. Statistical significance of comparison with the sample without incubation (0 h) was calculated by ANOVA; *, *p* < 0.05; #, *p* < 0.01; +, *p* < 0.005; **, *p* < 0.001.

Rabbit LDL, with or without pefabloc treatment, was incubated with copper sulfate for up to 8 h, and the profiles of PC molecular species were determined. Figures 5 and 6 show the time-course changes in peak area of each PC molecular species in rabbit oxLDL. The profile of S/MUFA-PC did not change throughout the incubation, and PUFA-PC appeared to decrease after 1 h (Figures 5A, 5B, 6A, and 6B). LysoPC increased during oxidation of rabbit LDL but it did not when rabbit LDL was pretreated with PAF-AH inhibitor (Figures 5C, 6C). These results, together with those from human oxLDL, indicate that the contribution of PAF-AH is required for lysoPC formation during oxidative modification of LDL. Many of the oxPC species increased their peak areas during the oxidation, however the major species accumulated in rabbit oxLDL, PONPC and long-chain oxPC with two double bonds, were the same as those in human oxLDL (Figures 5D, 5E, 6D and 6E).

**Figure 7:**
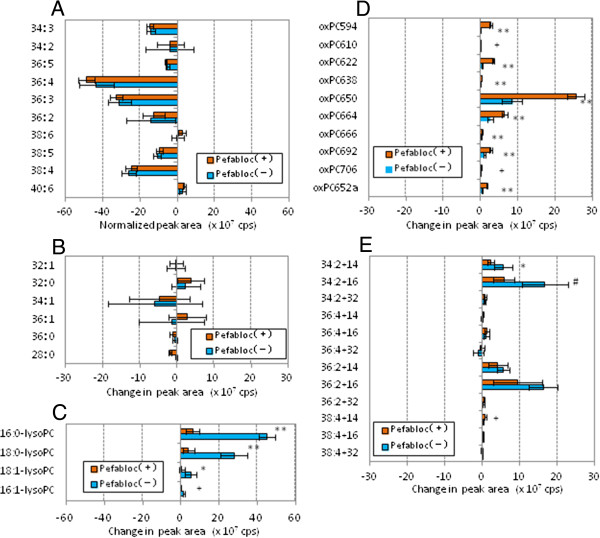
**Effects of PAF-AH inhibitor pretreatment on changes in PC species during oxidation of rabbit LDL.** Rabbit LDL and pefabloc-treated rabbit LDL (0.1 mg/mL) were incubated with copper sulfate (5 μM) at 37°C for 8 h. Lipid extraction and LC-MS/MS analysis were performed, and the change in peak area (cps) during the 8 h oxidation was calculated for each PC species. The 42 PC species were classified into five groups: PUFA-PC **(A)**, S/MUFA-PC **(B)**, lysoPC **(C)**, cleaved-chain oxPC **(D)** and long-chain oxPC **(E)**. Values indicated are means and SD (n = 6). Statistical significance between the samples with and without treatment with PAF-AH inhibitor was calculated by Welch t-test; *, *p* < 0.05; #, *p* < 0.01; +, *p* < 0.005; **, *p* < 0.001.

Figure 7 shows the change of peak area for each species after the 8 h incubation of rabbit LDL with or without pefabloc treatment. It was observed again that pretreatment of LDL with pefabloc had no effect on the behavior of PUFA-PC and S/MUFA-PC during the oxidation of rabbit LDL, which is very similar to human LDL (Figure 7A, B). LysoPC increased their peak areas during the oxidation reaction, and the lysoPC formation was suppressed in pefabloc-treated LDL (Figure 7C). Pefabloc treatment had enhanced the accumulation of cleaved oxPC species and suppressed long-chain oxPC species with two double bonds (Figure 7D, E).

### Protein modification with oxPC

**Figure 8:**
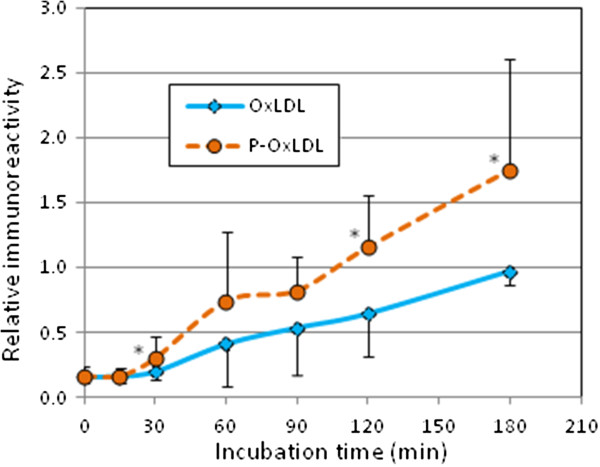
**Immunoreativity of human oxLDL to antibodies against oxPC and apoB.** Human LDL (0.2 mg/mL) treated either with or without a PAF-AH inhibitor, pefabloc, was incubated with copper sulfate (5 μM) at 37°C for up to 3 h. Oxidative changes of immunoreactivity by sandwich ELISA using antibodies against oxPC and apoB were evaluated. Relative immunoreactivity was calculated from 10 experiments. Statistical significance between samples with and without pefabloc treatment was calculated by Welch *t*-test; *, p < 0.05.

During oxidation of LDL, apoB-oxPC adducts are formed and can be detected by sandwich ELISA using antibodies recognizing oxPC and apoB [36]. Inhibition of PAF-AH activity in human LDL increased apoB-oxPC adducts formed in oxLDL, as judged from immunoreactivity in sandwich ELISA (Figure 8). PAF-AH hydrolyzes oxPC species with short and hydrophilic acyl chains including chemically reactive aldehyde derivatives [26]. We accordingly speculate that PAF-AH is protective to apoB-oxPC adduct formation.

## Discussion

OxLDL is thought to be an important factor in atherosclerosis. Evidence is accumulating that the plasma level of *in vivo* oxLDL is a predictor of cardiovascular disease [4-8]. Previous studies revealed that many atherogenic oxPCs and lysoPC are formed in oxLDL [12,15,18,20]. However, the manner in which LDL is oxidatively modified remains to be determined. In this study we analyzed the time-course changes in the PC profile during LDL oxidation using LC-MS/MS to view the full picture of oxPC generation and the contribution of PAF-AH. Our study shows that PAF-AH has a crucial role in generating lysoPC during LDL oxidation. Many cleaved oxPC and long-chain oxPC species are generated, but the data show only a few major oxPC species accumulating in oxLDL.

By simultaneously and selectively detecting 42 PC species in a sample using MRM mode of LC-MS/MS, we followed changes in PC profiles during copper-induced oxidation. Because standards for all of the PC species were not available, absolute quantitation of the PC profiles cannot be completed. The data are shown as the relative peak areas of the PC species after normalizing on the basis of internal standard. As expected, most of the PUFA-PC species declined sharply and some of them reduced largely, whereas S/MUFA-PC did not change. Long-chain oxPC with four double bonds were unstable and appeared transient. Interestingly, only a few oxPC species accumulated in fully oxidized LDL *in vitro*, namely PONPC (m/z = 650.6) and mono-oxygenated forms of linoleate-containing PC. It is likely that oxPC species derived from PC containing more than four double bonds are so susceptible to further modification either by chemically or enzymatically that they cannot accumulate in oxLDL.

PCOOH, detected as long chain oxPC species with m/z +32, was present in LDL but was not the major products in oxLDL (Figures 2, 3, 4, 5, 6 and 7, and Additional file 1 for enlarged graphs). Kinoshita, *et al*. determined the PCOOH concentration in human plasma from healthy control was 160 nmol/L using HPLC with chemiluminescence detector [37]. PCOOH concentration increased to approximately 2-fold in plasma from patients with hyperlipidemia, however, the ratio of PCOOH in total PC was still 1/7,000. Our data cannot directly transfer to quantitative calculations, but relatively small peaks for PCOOH in the human and rabbit LDLs among all the PC species (Figures 2 and 5) corresponds well to the previous study.

LysoPC appeared to be the major product in oxLDL. Its generation was strongly suppressed by pefabloc treatment of both human and rabbit LDL, suggesting that PAF-AH activity is critical for lysoPC generation in LDL. It is well known that PAF-AH can act on PC species with hydrophilic short chain acyl groups, in addition to PAF, to produce lysoPC [26]. Thus it is speculated that pefabloc treatment of LDL increases some oxPC species that are substrates of PAF-AH during oxLDL formation. The peak areas for several cleaved oxPC species further increased by the pefabloc treatment (Figures 4D and 7D). However, the major oxPC species that accumulated in pefabloc-treated LDL during oxidation was almost the same as that of oxLDL with active PAF-AH. Our observation agrees with a previous report by Davis *et al*., in which an oxPC profile in LDL oxidized with copper sulfate for 20 h was analyzed [35].

In addition to hydrolysis of cleaved oxPC species, the protective role of PAF-AH in oxLDL modification was suggested by oxPC-apoB adduct formation. Some chemically active oxPC may react with proteins to form adducts, and extensive hydrolysis of oxPC by PAF-AH is protective against apoB modification by oxPC products.

LysoPC generation was largely inhibited in pefabloc-treated oxLDL, but still a very small increase in lysoPC remained. A possible explanation for this observation is non-enzymatic hydrolysis of oxPC. Choi, *et al*. reported that lysoPC can be generated through spontaneous deacylation of cleaved-chain oxPC products such as 1-palmityl-2-(4-hydroxy-7-oxo-5-heptenoyl)-PC [38]. Alternatively, other oxPC-hydrolyzing enzymes may distribute in part to LDL and contribute to lysoPC formation. We propose that most of the oxPC species generated are not only hydrolyzed by PAF-AH but also further modified or decomposed in PAF-AH-independent manners.

Inhibition of PAF-AH in apoE-knockout mice resulted in reduction in atherosclerotic lesion size [39]. However, another study reported that adenoviral overexpression of PAF-AH prevents injury-induced neointima in apoE-knockout mice [40]. A PAF-AH inhibitor has been investigated in clinical trials and the prevention of expansion of necrotic core lesions in human was shown, however, the roles of PAF-AH in atherogenesis and oxLDL modification remain uncertain [41]. Our study suggests that PAF-AH inhibition decreased lysoPC formation but had little effect on oxPC accumulation. To understand the effects of PAF-AH on atherosclerosis, the pathological roles of atherogenic oxPC and lysoPC should be elucidated.

It should be noted that the current study focused on oxLDL prepared *in vitro*. This study is an important step toward understanding the complex nature of oxidized lipoproteins. Elucidation of the contribution of oxLDL to atherogenesis awaits further lipidomic and proteomic studies to characterize the features of circulating oxLDL.

## Conclusions

We determined the PC profile of oxLDL using a lipidomic approach. The major products in copper-induced oxLDL were lysoPC species, and PAF-AH plays a critical role in lysoPC generation in LDL. Although many oxPC species are generated, PONPC and long-chain oxPC species derived from linoleate-containing PC are the major products accumulating in oxLDL. These observations shed light on the manner in which oxPCs are modified and metabolized.

## Methods

### Preparation and oxidation of human LDL

Human LDL and oxLDL were prepared as described previously [9]. This study was approved by the Ethical Committee of Showa University. In brief, plasma was separated from human blood from healthy volunteers by centrifugation at 2,000 rpm for 10 min, after which EDTA was added to the plasma (final concentration 0.25 mM). LDL was separated from the human plasma using sequential ultracentrifugation, with addition of KBr to adjust the density, and then dialyzed against PBS containing 0.25 mM EDTA to remove the KBr. The protein concentration of the LDL fraction was determined by the BCA method using BSA as standard.

To inhibit PAF-AH activity of LDL, an aliquot of the LDL fraction (1 mg/mL) was incubated with pefabloc (Boehringer, 0.5 mM) at 37°C for 30 min [30]. The LDL was then passed through a PD-10 desalting column (BioRad) to remove excess pefabloc. The PAF-AH activity was estimated using a PAF-AH assay kit (Cayman Chemicals).

Either LDL or pefabloc-treated LDL (0.2 mg/mL) was incubated with CuSO_4_ (5 μM) at 37°C for up to 3 h. At the end of the incubation period, 1 μL of 250 mM EDTA was added to stop copper-induced oxidation. Oxidation reaction was evaluated by measurement of TBARS and agarose-gel electromobility assay. After mixtures of 100 μL of sample and 200 μL of TBA reagent (0.375% TBA, 15% trichloroacetic acid, and 0.25 M HCl) were boiled for 15 min, absorbance was measured at 535 nm [42]. Electromobility of oxLDL was evaluated on a 0.5% agarose gel using barbiturate buffer at pH 8.6. Determination of immunoreactivity to an anti-oxPC monoclonal antibody and an anti-apoB polyclonal antibody was performed by sandwich ELISA as described previously [36].

### Preparation and oxidation of rabbit LDL

New Zealand White rabbits (6–7 weeks old) were purchased from Kitayama Labes Co., Ltd. (Nagano, Japan). The study protocol was approved by the Animal Care Committee (permit no. 11–0221) and carried out in accordance with the “Guideline for Animal Care and Use at the Otsuka Pharmaceutical Co. Ltd.”. The rabbits were acclimated and fed a cholesterol-enriched diet containing 0.5% cholesterol (w/w) in a standard rabbit chow (RC4; Oriental Yeast Co. Ltd.) at 80 g/day for 10 weeks as previously described [43]. Blood was collected from the inferior vena cava in EDTA-treated syringes under pentobarbital anesthesia. Then LDL fraction was collected from plasma by centrifugation, 0.25 mM EDTA was added, and LDL was stored in tubes filled with argon gas in a refrigerator. After LDL was dialyzed against PBS in a refrigerator, 5 μM CuSO_4_ was added to 100 μg/mL of LDL and incubated for up to 8 h at 37°C. The oxidation reaction was stopped by addition of EDTA (final 0.25 μM) to the solution. The oxLDL was stored in tubes filled with argon gas at -80°C.

### LC-MS/MS analysis of oxPC

The profile of PC molecular species in LDL and oxLDL was determined by LC-MS/MS (AB SCIEX, QTRAP5500 equipped with Shimadzu LC-10A). LC separations were performed on an Inertsil SIL-100A column (2.1 × 150mm, GL Science, Tokyo, Japan) with elution solvent of acetonitrile/methanol/3% formic acid (18/11/8 (v/v/v)) at a flow rate 200 μL/min as described by Morishita *et al.*[44]. The synthetic PC standards, didecanoyl-PC, 1-pentadecanoyl-2-lysoPC, PONPC were purchased from Avanti Polar Lipid Inc. Total lipids were extracted from 10 μg of oxLDL by the Bligh and Dyer method [45]. To the recovered chloroform phase, didecanoyl-PC (20 pmol) was added as internal standard. The samples were dried under N_2_ gas and the dissolved in 200 μL of the elution solvent. For each analysis, 10 μL of the sample was injected. PC species were detected and quantified using MRM procedure, in which 42 selected ions releasing a fragment ion of m/z = 184 corresponding to a phosphorylcholine group were detected.

### Statistics

Data are presented as means ± standard deviation (SD). For the statistical analysis, one-way-ANOVA was carried out, and a *p* value < 0.05 was taken to be significant.

## Abbreviations

apoB: Apolipoprotien B; cleaved oxPC: oxPC with truncated acyl chains; ELISA: Enzyme-linked immunosorbent assay; HDL: High-density lipoprotein; LC-MS/MS: Liquid chromatography-tandem mass spectrometry; LDL: Low-density lipoprotein; long-chain oxPC: oxPC with oxidized acyl chains; lysoPC: Lysophosphatidylcholine; MRM: Multiple-reaction monitoring; MUFA: Monounsaturated fatty acids; oxLDL: Oxidized low-density lipoprotein; oxPC: Oxidized phosphatidylcholine; PAF-AH: Platelet-activating factor acetylhydrolase; PC: Phosphatidylcholine; PCOOH: PC hydroperoxide; PONPC: 1-palmitoyl-2-(9-oxononanoyl) PC; PUFA: Polyunsaturated fatty acids; PUFA-PC: PC with polyunsaturated fatty acids; SD: Standard deviation; S/MUFA-PC: PC with saturated or monounsaturated fatty acids; TBARS: Thiobarbituric acid-reactive substances.

## Competing interests

The authors declare that they have no competing interest.

## Authors’ contribution

NS, YK and HI designed the study. NS, YK, TO, NI, YM, YI, TA, RK, ToYa, HK, and SI performed the experiments and NS, YK and HI analyzed the data. HI and YK wrote the manuscript. ToYa, AM, SH and ToYo performed critical reading of the manuscript and discussion. All authors read and approved the final manuscript.

## Supplementary Material

Additional file 1 **Enlarged figures for PC profile changes during copper-induced oxidation of human and rabbit LDL.** Some bar graphs of the PC species shown in the Figures 2, 3, 5 and 6 were too small to see (less than 1 × 10^7^ cps), this file shows the detail of the PC profile changes of the minor species. The time course changes of PC species during oxidation of human LDL with or without pefabloc pretreatment (A, B) are shown in an enlarged scale. The time course changes of ten PC species during oxidation of rabbit LDL with or without pefabloc (C, D) are shown in an enlarged scale. Statistical significance of comparison with the sample without incubation (0 min) was calculated by ANOVA; *, *p* < 0.05; #, *p* < 0.01; +, *p* < 0.005; **, *p* < 0.001. Click here for file
