# Supplementary figures and images for: Time course-changes in phosphatidylcholine profile during oxidative modification of low-density lipoprotein
Source: Lipids Health Dis. 2014 Mar 14;13:48. doi: 10.1186/1476-511X-13-48 (PMC4007754; doi:10.1186/1476-511X-13-48)

Additional file 1

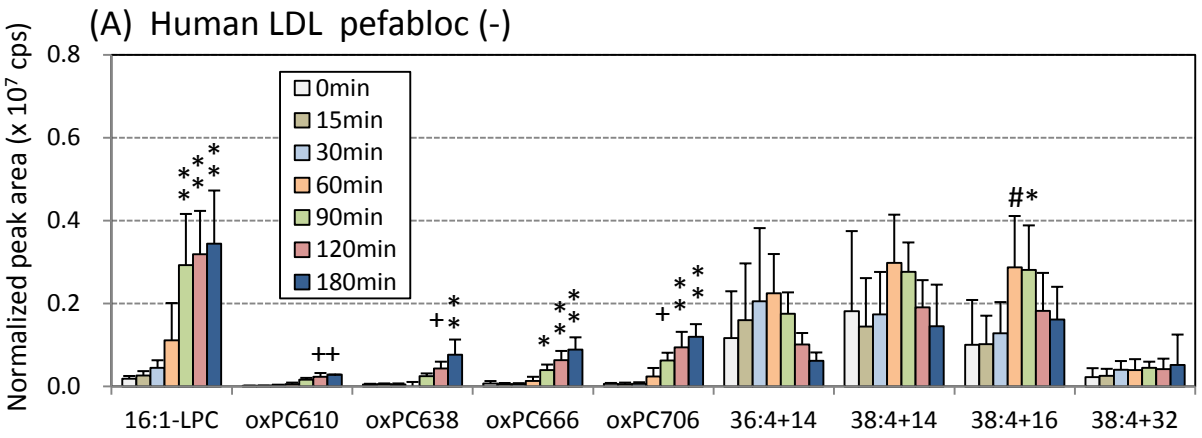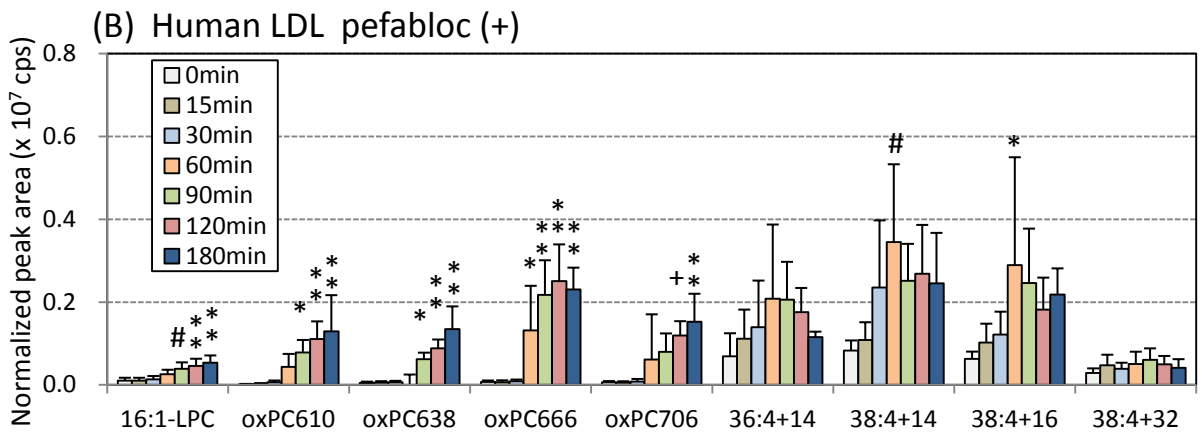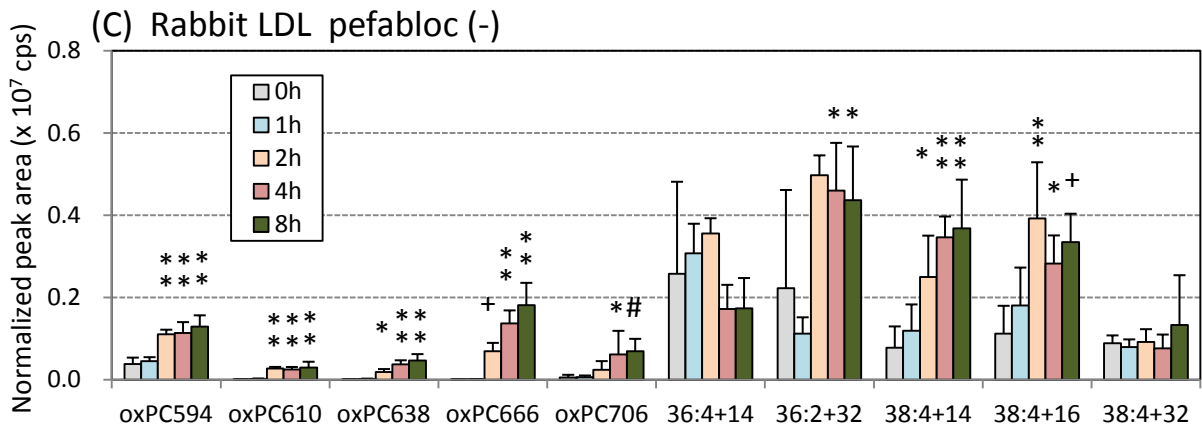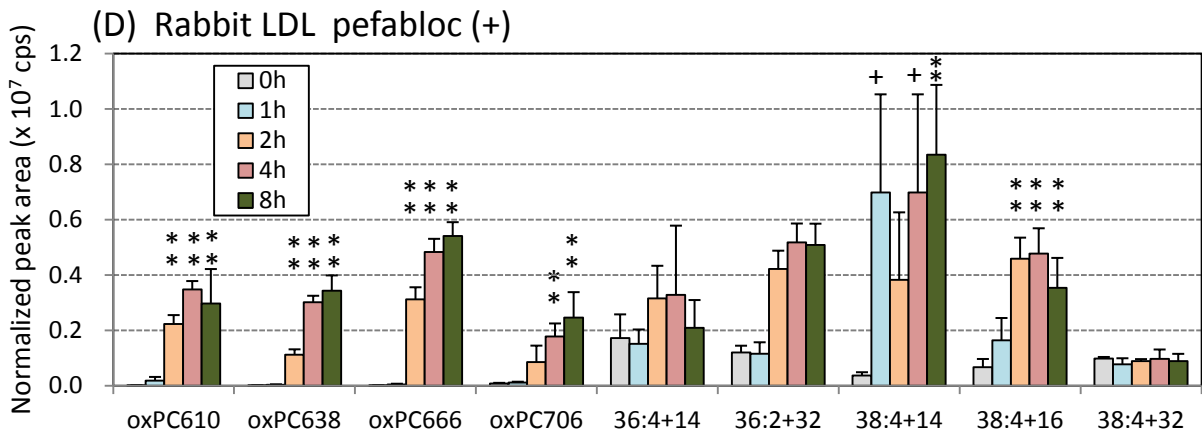

Supplement: Additional file 1 — Enlarged figures for PC profile changes during copper-induced oxidation of human and rabbit LDL. Some bar graphs of the PC species shown in the Figures 2, 3, 5 and 6 were too small to see (less than 1 × 10^7^ cps), this file shows the detail of the PC profile changes of the minor species. The time course changes of PC species during oxidation of human LDL with or without pefabloc pretreatment (A, B) are shown in an enlarged scale. The time course changes of ten PC species during oxidation of rabbit LDL with or without pefabloc (C, D) are shown in an enlarged scale. Statistical significance of comparison with the sample without incubation (0 min) was calculated by ANOVA; *, p < 0.05; #, p < 0.01; +, p < 0.005; **, p < 0.001. [file 1476-511X-13-48-S1.pdf]
